# Joint haplotype assembly and genotype calling via sequential Monte Carlo algorithm

**DOI:** 10.1186/s12859-015-0651-8

**Published:** 2015-07-16

**Authors:** Soyeon Ahn, Haris Vikalo

**Affiliations:** Department of Electrical and Computer Engineering, The University of Texas at Austin, Austin, 78712 Texas USA

**Keywords:** Haplotype assembly, Deterministic sequential Monte Carlo, Genotype calling

## Abstract

**Background:**

Genetic variations predispose individuals to hereditary diseases, play important role in the development of complex diseases, and impact drug metabolism. The full information about the DNA variations in the genome of an individual is given by haplotypes, the ordered lists of single nucleotide polymorphisms (SNPs) located on chromosomes. Affordable high-throughput DNA sequencing technologies enable routine acquisition of data needed for the assembly of single individual haplotypes. However, state-of-the-art high-throughput sequencing platforms generate data that is erroneous, which induces uncertainty in the SNP and genotype calling procedures and, ultimately, adversely affect the accuracy of haplotyping. When inferring haplotype phase information, the vast majority of the existing techniques for haplotype assembly assume that the genotype information is correct. This motivates the development of methods capable of joint genotype calling and haplotype assembly.

**Results:**

We present a haplotype assembly algorithm, ParticleHap, that relies on a probabilistic description of the sequencing data to jointly infer genotypes and assemble the most likely haplotypes. Our method employs a deterministic sequential Monte Carlo algorithm that associates single nucleotide polymorphisms with haplotypes by exhaustively exploring all possible extensions of the partial haplotypes. The algorithm relies on genotype likelihoods rather than on often erroneously called genotypes, thus ensuring a more accurate assembly of the haplotypes. Results on both the *1000 Genomes Project* experimental data as well as simulation studies demonstrate that the proposed approach enables highly accurate solutions to the haplotype assembly problem while being computationally efficient and scalable, generally outperforming existing methods in terms of both accuracy and speed.

**Conclusions:**

The developed probabilistic framework and sequential Monte Carlo algorithm enable joint haplotype assembly and genotyping in a computationally efficient manner. Our results demonstrate fast and highly accurate haplotype assembly aided by the re-examination of erroneously called genotypes.

A C code implementation of ParticleHap will be available for download from https://sites.google.com/site/asynoeun/particlehap.

## Background

Increased affordability of high-throughput DNA sequencing has enabled studies of genetic variations and of the effects they have on health and medical treatments. In diploid organisms, such as humans, chromosomes come in pairs. The chromosomes in a pair of autosomes are homologous, i.e., they have similar composition and carry the same type of information but are not identical. The most common type of DNA sequence variation is a single nucleotide polymorphism (SNP), where a single base in the genome differs between individuals or paired chromosomes. Each of those variants is referred to as an allele; a SNP has at least two different alleles. If the two alleles at a SNP site are same, the SNP site is homozygous; if they are different, it is heterozygous. SNP calling is concerned with identification of the locations and types of such alleles, and is followed by genotype calling to decide the genotypes associated with the locations of the detected SNPs. Accurate SNP and genotype calling are challenging due to uncertainties caused by base calling and read alignment errors. The low-to-medium coverages typical of large-scale sequencing projects are often associated with erroneous SNP and genotype calling [1]. As an illustration, in the low-coverage (2−6×) 1000 Genomes Project pilot, the genotype accuracy at heterozygous sites was 90 % for the lowest allele frequencies (minor allele frequency (MAF) <3 *%*), 95 % for the intermediate frequencies (MAF 50 %), and 70-80 % for the highest frequency variants (MAF >97 *%*) [2].

SNP and genotype calling do not assign alleles to specific chromosomes in the pairs. Such detailed information is provided by haplotypes, ordered collections of SNPs on the chromosomes. Haplotypes have been of fundamental importance for the studies of human diseases and effectiveness of drugs [3]. The International Haplotype Map Project’s pursuit of developing a haplotype map of the human genome reflects the significance of acquiring and understanding haplotype information [4]. *Haplotype inference* typically refers to the task of reconstructing haplotypes from the genotype samples of a population. *Haplotype assembly*, or single individual haplotyping, aims to reconstruct single individual haplotypes from high-throughput sequencing data. Since the SNP sites are assumed to be bi-allelic (i.e., each SNP site contains one of only two possible nucleotides), the alleles are labelled as 0 and 1 and the haplotypes are represented by binary sequences. Therefore, haplotype assembly is often cast as the problem of phasing two binary sequences from their short samples (i.e., reads) that are represented by ternary strings (where the third symbol denotes missing information). Majority of the existing haplotype assembly algorithms rely on this formulation of the problem [5].

Several haplotype assembly criteria and algorithms to optimize them were considered in [6, 7]. The minimum error correction (MEC) criterion, in particular, has received a considerable amount of attention and has been broadly used in practice. Most of the haplotype assembly problem formulations have been shown to be NP-hard [6–8], which has motivated numerous computationally efficient heuristic solutions [5]. FastHare, proposed in [9], was an early heuristic method that was followed by several approximate techniques in [10, 11]. In [12, 13], the use of clustering approaches for splitting reads into two sets, each associated with one chromosome in a pair, was proposed. In addition to the approximate methods, several algorithms that search for the exact solution to the MEC formulation of the problem were developed, including the branch-and-bound technique in [14]. However, as argued in [15], the exact algorithms are often infeasible in practice; the approach in [16] based on the Markov Chain Monte Carlo (MCMC) method, HASH, also incurs high computational cost while being more accurate than heuristics. As a follow-up to HASH, [17] presented a significantly faster heuristic algorithm, HapCUT, suffering only a minor loss of accuracy. To minimize the MEC score, HapCUT iteratively computes the max-cut in a graph that represents the assembly problem. In [18], another max-cut based heuristic, ReFHap, was proposed; ReFHap relies on a different graph structure to achieve higher speed while maintaining accuracy similar to that of HapCUT. Other methods include a dynamic programming solution in [19]; a method that solves an appropriate integer linear program [20]; and several other heuristics including [21], H-BOP [22], HMEC [23], and HapCompass [24, 25].

A probabilistic framework for haplotype assembly was first introduced in [26]. There, in order to deal with inherently random errors in sequencing data, the probability that a site in the fragments is incorrectly sequenced is defined for each of the four nucleotide bases. The most likely haplotype phases between SNP sites are determined using joint posteriori probabilities whose calculation is limited to two or three adjacent SNPs due to the intensive computational cost that grows exponentially in the number of SNP sites. The locally estimated haplotype segments are linked if the corresponding confidence levels exceed a certain threshold. In the follow-up work [27], reconstruction of longer haplotype segments using the Gibbs sampling procedure was enabled. However, this iterative approach still first assembles short haplotype segments that are then connected, and requires runtimes infeasible for block lengths typically encountered in practice. More recently, [28] proposed a new probabilistic mixture model, MixSIH, which leads to a more efficient computation of the haplotype likelihoods than those in [26, 27]. However, MixSIH is still about 10-fold slower than either HapCUT [17] or ReFHap [18] while having comparable accuracy, and the model there is restricted to the bi-allelic representation as in [6-25].

It is worth pointing out that most of the existing algorithms for haplotype assembly allow no more than two alleles at a SNP site and only deal with the errors caused by substitutions between those two alleles [6-25,28]. In practice, when sequencing errors lead to reads that report more than two alleles at a SNP site, either all of the tri- or tetra-allelic sites are discarded [10, 17] or the alleles that do not match the reference (or its alternative) are thrown away [24, 25]. The former drastically reduces not only the number of SNP sites to be reconstructed but also the chance of reliable full haplotype reconstruction (due to reducing the length of already short reads). The main drawback to the latter is that by fully trusting genotype information provided by SNP/genotype calling, the true haplotypes may be incorrectly reconstructed when the genotype calling is erroneous (i.e., when alleles corresponding to an incorrect genotype are preserved while the alleles corresponding to the true genotype are discarded).

In this paper, we propose a novel method that relies on a probabilistic model of the data to incorporate genotype calling in the haplotype assembly procedure. Unlike [26, 27], the proposed method infers both the most likely genotypes and haplotype phases by examining the complete set of SNP loci in a computationally efficient manner. To this end, we employ the sequential Monte Carlo (SMC) algorithm (i.e., a particle filter). Particle filters are capable of sequentially estimating the posterior density of unknown variables by representing them with a set of particles and associated weights [29]. When the solution space is discrete and finite, a deterministic form of SMC can be derived [30, 31]; this has been exploited for solving various problems in genomics [32, 33]. Noting that the set of possible haplotype pairs is discrete and finite, we develop a modified deterministic sequential Monte Carlo (DSMC) method for solving the haplotype assembly problem. Our algorithm, ParticleHap, relies on the 2 ^*nd*^-order Markov model of the haploype sequence to search for the most likely association of the SNPs to haplotypes. Phasing of the SNPs is done sequentially: the posteriori probability of a partial haplotype comprising *n* SNPs is calculated using the read information about the SNP in the *n* ^*th*^ position of the haplotype sequence and the posteriori probability of the previously inferred (*n*−1)-bases long partial haplotype. By working with SNP calls rather than their binary representations (the latter is typically used by state-of-the-art haplotype assembly algorithms), ParticleHap can reliably infer the most likely genotypes. Our extensive computational studies demonstrate that the proposed scheme is more accurate and computationally more efficient than state-of-the-art methods in [17, 18].

## Methods

### Problem formulation

In our model and the subsequently proposed haplotype assembly algorithm, we focus on the SNP sites where two or more alleles are observed. The sites with only one observed allele are declared homozygous and not used for the assembly. Assume there are *m* paired-end short reads covering *n* remaining SNP sites. Such data can be represented by an *m*×*n* matrix where the rows contain information provided by the reads while the columns correspond to the SNP sites.

By adopting the notation used in [26, 27], let **X** with elements $X_{\textit {ij}}=x_{\textit {ij}}, x_{\textit {ij}}\in \mathcal {B}$, be the matrix of potentially erroneous observations, while **Y** with entries $Y_{\textit {ij}}=y_{\textit {ij}}, y_{\textit {ij}}\in \mathcal {A}$, denote the corresponding error-free data matrix, 1≤*i*≤*m*, 1≤*j*≤*n*, $\mathcal {A}=\{\mathsf {A},\mathsf {C},\mathsf {G},\mathsf {T}\}$ and $\mathcal {B}=\{\mathsf {A},\mathsf {C},\mathsf {G},\mathsf {T},-\}$. Here − denotes a gap, i.e., a site not covered by a read or an ambiguous base-call. Let a 2×*n* matrix **S** with entries *S*_*kj*_=*s*_*kj*_, $s_{\textit {kj}}\in \mathcal {A}$, *k*∈{1,2}, denote the true haplotype pair, and let us collect the indicators of the origin of the reads, *f*_*i*_∈{1,2}, 1≤*i*≤*m*, into a vector **F**. With this notation, the true bases relate to the true haplotypes as $\phantom {\dot {i}\!}Y_{\textit {ij}}=S_{f_{i},j}$, where 1≤*i*≤*m*, 1≤*j*≤*n*. We assume that the composition probabilities Pr(*S*_*kj*_=*s*), $s\in \mathcal {A}$, at each SNP position are mutually independent and constant across haplotypes. The measurement model is given by $ \text {Pr}(X_{\textit {ij}}=x_{\textit {ij}} | Y_{\textit {ij}}=y_{\textit {ij}}), \; y_{\textit {ij}}\in \mathcal {A}, \; x_{\textit {ij}}\in \mathcal {B}$. A sequencing error occurs when the true base *Y*_*ij*_=*y*_*ij*_ is misread as *X*_*ij*_=*x*_*ij*_, *x*_*ij*_≠*y*_*ij*_. Note that the posteriori probability *p*(**S**|**X**) can be computed from *p*(**S**,**X**) and $p(\mathbf {X})=\sum \limits _{\mathbf {S}}\displaystyle {p(\mathbf {S},\mathbf {X})}$ using the Bayes’ rule, where

(1) $$\begin{array}{@{}rcl@{}} p(\mathbf{S},\mathbf{X}) &=& \left(\frac{1}{2}\right)^{m}\prod\limits_{j=1}^{n}p(S_{1j})\prod\limits_{j=1}^{n}p(S_{2j}) \\ &\: \times&\prod\limits_{i=1}^{m}\left[\prod\limits_{j=1}^{n}p(X_{i j} \mid S_{1 j}) +\prod\limits_{j=1}^{n}p(X_{i j} | S_{2 j})\right]. \end{array} $$

We assume that each read is generated from one of the two haplotypes with probability $\frac {1}{2}$, i.e., $\text {Pr}(f_{i}=1)=\text {Pr}(f_{i}=2)=\frac {1}{2}$.

### The ParticleHap algorithm

Following the adopted notation, the goal of haplotype assembly is to determine matrix **S** from the observation matrix **X**. A Bayesian approach to solving this problem involves maximization of the posteriori distribution *p*(**S**|**X**). Let *S*_·*j*_ and *X*_·*j*_ denote the *j* ^*th*^ column vectors of **S** and **X**, respectively, and let us define S_1:*t*_={*S*_·1_,*S*_·2_,⋯,*S*_·*t*_} and X_1:*t*_={*X*_·1_,*X*_·2_,⋯,*X*_·*t*_}. Recursive Bayesian estimation (i.e., Bayesian filtering) is concerned with recursively finding the conditional probability density function *p*(S_1:*t*_|X_1:*t*_). Having obtained the estimate $\hat {p}(\mathrm {S}_{1:t} | \mathrm {X}_{1:t})$, we can determine the most likely S_1:*t*_. However, finding an analytical form of this probability density function is often infeasible, as is the case for the haplotype assembly problem.

Sequential Monte Carlo (SMC), often referred to as particle filtering [29], describes *p*(S_1:*t*_|X_1:*t*_) using a set of discrete points (particles) and their corresponding weights. SMC can be interpreted as the dynamical system which, in the context of haplotype assembly, comprises the initial state model *p*(*S*_·1_), state transitions model *p*(*S*_·*t*_|*S*_·*t*−1_) and measurement model *p*(*X*_·*t*_|*S*_·*t*_). The distribution *p*(S_1:*t*_|X_1:*t*_) can be propagated using an importance sampling technique where samples from a proposal density *q*(S_1:*t*_|X_1:*t*_) are generated and appropriately weighted. Having drawn *K* samples $\left \{S_{\cdot t}^{(1)}, S_{\cdot t}^{(2)},\cdots,S_{\cdot t}^{(K)}\right \}$ from *q*(S_1:*t*_|X_1:*t*_) and assigned them weights $w_{t}^{(k)}$, *p*(S_1:*t*_|X_1:*t*_) can be approximated by

(2) $$ {\small{\begin{aligned} {}\hat{p}(\mathrm{S}_{1:t} | \mathrm{X}_{1:t}) = \frac{1}{W_{t}}\sum\limits_{k=1}^{K}w_{t}^{(k)}\delta\left(\mathrm{S}_{1:t}-\mathrm{S}_{1:t}^{(k)}\right), \; w_{t}^{(k)}= \frac{p(\mathrm{S}_{1:t} | \mathrm{X}_{1:t})} {q(\mathrm{S}_{1:t} | \mathrm{X}_{1:t})}, \end{aligned}}} $$

where $W_{t}=\sum \limits _{k=1}^{K}w_{t}^{(k)}$ and *δ*(·) is an indicator function, i.e., *δ*(*s*−*s*_0_)=1 for *s*=*s*_0_ and *δ*(*s*−*s*_0_)=0 otherwise. The weight $w_{t}^{(k)}$ can be further derived as ([29])

(3) $$\begin{array}{@{}rcl@{}} w_{t}^{(k)} & \propto & w_{t-1}^{(k)}p\left(X_{\cdot t} \mid S_{\cdot t-1}^{(k)}\right)  \\ & \propto & w_{t-1}^{(k)} \sum\limits_{l=1}^{L}p(X_{\cdot t} \mid S_{\cdot t}=s_{l})p\left(S_{\cdot t}=s_{l} \mid S_{\cdot t-1}^{(k)}\right). \end{array} $$

In the traditional SMC, the set $\left \{\left (\mathrm {S}_{1:t}^{(k)},w_{t}^{(k)}\right), k=1,\cdots,K{\vphantom {\mathrm {S}_{1:t}^{(k)},w_{t}^{(k)}}}\right \}$ is recursively generated from the previous set of properly weighted samples $\left \{\left (\mathrm {S}_{1:t-1}^{(k)},w_{t-1}^{(k)}\right), k=1,\cdots,K{\vphantom {\mathrm {S}_{1:t-1}^{(k)},w_{t-1}^{(k)}}}\right \}$ by using the optimal proposal distribution $q\left (S_{\cdot t} | \mathrm {S}_{1:t-1}^{(k)},\mathrm {X}_{1:t}\right) = p\left (S_{\cdot t} | \mathrm {S}_{1:t-1}^{(k)},\mathrm {X}_{1:t}\right)$,

(4) $$ {}q\left(S_{\cdot t}=s_{l} | \mathrm{S}_{1:t-1}^{(k)},\mathrm{X}_{1:t}\right) \propto p(X_{\cdot t} | S_{\cdot t}=s_{l}) p\left(S_{\cdot t}=s_{l} | S_{\cdot t-1}^{(k)}\right).  $$

In contrast to the conventional SMC, the *deterministic sequential Monte Carlo* (DSMC, [30, 31]) explores all possible states in each step of the recursive procedure. In particular, each particle at step *t*−1, $S_{\cdot t-1}^{(k)}, k=1,\cdots,K,$ is propagated to *L* possible states at step *t* instead of being propagated to a single particle, where *L* denotes the number of possible extensions of the partially reconstructed haplotype sequence in our haplotype assembly problem. Maintaining and further propagating all such particles would inevitably increase their number exponentially; to remedy this problem, in each step only *K* particles with the highest weights among *KL* possible particles are selected. Then, given a set $\left \{\left (\mathrm {S}_{1:t-1}^{(k)},w_{t-1}^{(k)}\right), k=1,\dots,K\right \}$ that does not contain duplicate paths, (2) and Bayes’ theorem lead to an approximation of the posterior distribution of S_1:*t*_

(5) $$ {}\hat{p}^{\text{\tiny DSMC}}(\mathrm{S}_{1:t} | \mathrm{X}_{1:t}) \,=\, \frac{1}{W_{t}^{\text{\tiny DSMC}}}\sum\limits_{k=1}^{K}\sum\limits_{l=1}^{L} w_{t}^{(k,l)} \delta\left(\mathrm{S}_{1:t}\,-\,\left[\mathrm{S}_{1:t-1}^{(k)} \; s_{l}\right]\right),  $$

where $W_{t}^{\text {\tiny DSMC}}=\sum \limits _{k,i} w_{t}^{(k,l)}$ and $\left [\mathrm {S}_{1:t-1}^{(k)},s_{l}\right ]$ is obtained by appending the state *s*_*l*_ to $\mathrm {S}_{1:t-1}^{(k)}$. Each weight $w_{t}^{(k,l)}$ is calculated as

(6) $$ w_{t}^{(k,l)} \propto w_{t-1}^{(k)} p(X_{\cdot t} | S_{\cdot t}=s_{l}) p\left(S_{\cdot t}=s_{l} | S_{\cdot t-1}^{(k)}\right).  $$

**Fig. 1:**
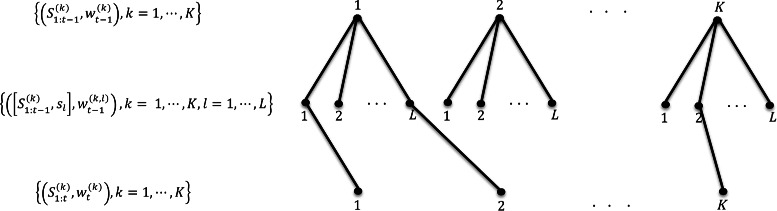
Procedure of propagating particles in deterministic sequential Monte Carlo. Each of *K* particles at step *t*−1 is propagated to *L* possible states at step *t*. Among *K*×*L* possible particles, only *K* particles with the highest weight are selected

The procedure is continued until obtaining $\mathrm {S}_{1:n}^{(k)}=\left (\mathrm {S}_{1:n-1}^{(k)},S_{\cdot n}^{(k)}\right)$ and its corresponding weights. Figure 1 illustrates the procedure of propagating particles in the DSMC. Each of *K* particles at step *t*−1 is propagated to *L* possible states at step *t*. Among *K*×*L* possible particles, only *K* particles with the highest weight are selected.

The conditional distribution *p*(*X*_·*t*_|*S*_·*t*_=*s*_*l*_) in the DSMC weight updates (6) reflects dependence of *X*_·*t*_ on the current state *S*_·*t*_ only, and does not include the phase information between nearby SNP sites (note that it does enable detection of the most likely genotypes at the *t* ^*th*^ site). To incorporate the phasing information, we extend the representation of the particle trajectories to the 2^*nd*^-order Markov model. In particular, we modify (6) so that the weight updates in our ParticleHap depend on the history of the state and the observation at *t*−1 as well as on the current state,

(7) $$\begin{array}{@{}rcl@{}} w_{t}^{(k,l)} & \propto & w_{t-1}^{(k)} p\left(X_{\cdot t} | S_{\cdot t}=s_{l},S_{\cdot t-1}^{(k)},X_{\cdot t-1}\right)  \\ & \times & p\left(S_{\cdot t}=s_{l} | S_{\cdot t-1}^{(k)},X_{\cdot t-1}\right), \end{array} $$

where $\phantom {\dot {i}\!}s_{l}=(s_{l_{1}},s_{l_{2}})$, *l*=1,…,*L*. In particular, at step *t*, ParticleHap examines potential extensions of the partially reconstructed haplotype by adding a single SNP site, which requires no more than 12 likelihood calculations – one for each possible heterozygous pair $(s_{l_{1}},s_{l_{2}})$ at site *t*, with 12 such pairs when there are 4 different bases in the *t* ^*th*^ column of **X**.

**Fig. 2:**
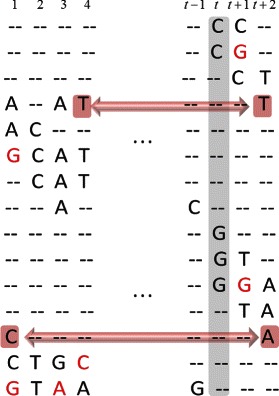
Information about heterozygous sites provided by paired-end reads and organized in the observation matrix X. Erroneous base characters are highlighted in red font

While the conditioning on $S_{\cdot t-1}^{(k)}$ and *X*_·*t*−1_ in $p\left (X_{\cdot t} | S_{\cdot t}=s_{l},S_{\cdot t-1}^{(k)},X_{\cdot t-1}\right)$ in (7) introduced phase information between SNPs in positions *t*−1 and *t*, there remains a major challenge for reconstruction of unknown haplotype due to gaps in the data matrix **X**. Even with the previously described 2^*nd*^-order Markov model of particle trajectories, phase information between two consecutive SNP sites cannot be retrieved using a read that is not covering both of those sites. For example, in Fig. 2, column *t*+2 contains 5 informative entries (i.e., entries which are not −). However, 2 of them belong to the reads which do not cover the SNP in the position *t*+1, i.e., are immediately preceded by −, and thus do not contribute to the phase information if the weights are computed according to (7). To this end, we modify (7) so that the information spread across gaps within paired-end reads can be utilized for generating particle trajectories. In particular, let us introduce a new variable $\mathbf {Pos}^{t} = \left \{po{s_{i}^{t}}, i = 1,\cdots,m, po{s_{i}^{t}} \in \{0,1,2,\cdots,t\}\right \}$, where $po{s_{i}^{t}}$ is the nearest informative (non-gap) position in the *i* ^*th*^ row left of the column *t*; note that ${pos}_{i}^{t+1} = po{s_{i}^{t}}$ if *X*_*i*,*t*+1_=−. Also, note that ${pos}_{i}^{t-1} = 0$ implies that there are no informative positions in the *i* ^*th*^ row left of the column *t* (i.e., *X*_*ij*_=− for all *j*≤*t*−1). With this notation, we rephrase (7) as

(8) $$\begin{array}{@{}rcl@{}} w_{t}^{(k,l)} & \propto & w_{t-1}^{(k)} p\left(X_{\cdot t} | S_{\cdot t}=s_{l},S_{\cdot \mathbf{Pos}^{t-1}}^{(k)},X_{\cdot \mathbf{Pos}^{t-1}}\right)  \\ & \times & p\left(S_{\cdot t}=s_{l} | S_{\cdot \mathbf{Pos}^{t-1}}^{(k)},X_{\cdot \mathbf{Pos}^{t-1}}\right). \end{array} $$

The measurement model in (8) assumes that the *i* ^*th*^ read is randomly generated from one of the two haplotypes, i.e.,

$$ \begin{aligned} p&\left(X_{\cdot t} | S_{\cdot t}=s_{l},S_{\cdot \mathbf{Pos}^{t-1}}^{(k)},X_{\cdot \mathbf{Pos}^{t-1}}\right)\\ &=\prod\limits_{i=1,x_{it}\neq -}^{m} p\left(X_{i,t} | S_{\cdot t}=s_{l},S_{\cdot {pos}_{i}^{t-1}}^{(k)},X_{i,{pos}_{i}^{t-1}}\right), \end{aligned} $$

where

(9) $$\begin{array}{@{}rcl@{}} && p\left(X_{i,t} | S_{\cdot t}=s_{l},S_{\cdot {pos}_{i}^{t-1}}^{(k)},X_{i,{pos}_{i}^{t-1}}\right) \\ &=&\left\{ \begin{array}{ll} p(x_{it} | s_{l_{1}}), & \text{if}~ X_{i,{pos}_{i}^{t-1}}=S_{1,{pos}_{i}^{t-1}}^{(k)},\\ p(x_{it} | s_{l_{2}}), & \text{if}~X_{i,{pos}_{i}^{t-1}}=S_{2,{pos}_{i}^{t-1}}^{(k)}\\ \frac{p(x_{it} | s_{l_{1}}) + p(x_{it} | s_{l_{2}})}{2}, & \textrm{otherwise.}\\ \end{array} \right. \end{array} $$

When computing $p\left (S_{\cdot t}=s_{l} | S_{\cdot \mathbf {Pos}^{t-1}}^{(k)}, X_{\cdot \mathbf {Pos}^{t-1}}\right)$, we assume that there is no correlation between the consecutive SNPs. Therefore, the state transition distribution is formed using the composition probabilities, e.g., $p\left (S_{\cdot t}=s_{l} | S_{\cdot \mathbf {Pos}^{t-1}}^{(k)},X_{\cdot \mathbf {Pos}^{t-1}}\right) =\text {Pr}(S_{1t}=s_{l_{1}})\text {Pr}(S_{2t}=s_{l_{2}})$. Note that, in principle, side information such as genotype frequencies or the patterns of linkage disequilibrium (LD) can be incorporated in state transition probabilities.

Going back to the example illustrated in Fig. 2, the modification of the weights shown in (8) now allows ParticleHap to retrieve phase information at position *t*+2 from the reads (highlighted in orange) that have a gap in position *t*+1 but cover some SNPs in the positions left of the (*t*+1)^*st*^ one. This often has a beneficial effect on the switch error rate, defined as the ratio of the number of SNP positions where the two chromosomes of a resulting haplotype phase must be switched in order to reconstruct the true phase. As an illustration, consider column *t* in the observation matrix shown in Fig. 2. Since none of the reads that cover SNPs at column *t* provide any phasing information, the partially reconstructed haplotype pair would equally likely be extended by either (C,G) or (G,C) which might lead to the switch error at the position *t*. This ambiguity is resolved at position *t*+2 from the reads *i*_1_=4 and *i*_2_=13 (highlighted in orange), which do not cover sites *t*+1, *t* nor *t*−1, but do cover sites 4 and 1, respectively: ParticleHap relying on those reads in (8)-(9) will assign larger weight to the particle propagated along the correct state path.

To initialize the algorithm at *t*=1 from *k* possible SNPs, all possible assignments are considered as $S_{\cdot 1}^{(k)}, k=1,\dots,K$, with the corresponding weights $w_{1}^{(l)}, l=1,\dots,L$, computed as $w_{1}^{(l)}\propto p(X_{\cdot 1} | S_{\cdot 1}^{(l)}=s_{l})$. From *t*=2, all possible extensions of the (*t*−1)-long haplotype are considered, and the extensions having non-zero weights are used to generate particles until *K* such particles are created. Once the set of *K* particles is formed, the subsequently generated particles are included in the set if their weight is greater than the weight of at least one particle that is already in the set; the latter then needs to be excluded from the set so that its cardinality remains *K*.

The ParticleHap algorithm is formalized below.

- **Step 1** (Initialization): For the first SNP position, compute $w_{1}^{(l)}\propto p\left (X_{\cdot 1} | S_{\cdot 1}^{(l)}=s_{l}\right)$, *l*=1,…,*L*. Normalize $w_{1}^{(l)}$ and store the corresponding possible haplotype pairs in $S_{\cdot 1}^{(k)}$, *k*=1,2,⋯,*K*.
- **Step 2** (Run iterations for 2≤*t*≤*n*): For each step *t*, *t*=2,…,*n*, enumerate all possible extensions of the existing particles $S_{\cdot t-1}^{(k)}$, thus generating $S_{\cdot t}^{(k,l)}=\left [S_{\cdot t-1}^{(k)},s_{l}\right ]$, *l*=1,⋯,*L*. For all *l*, compute the weights $w_{t}^{(k,l)}$ using (8).
- **Step 3** (Particle selection): Select and store *K* particles $\left \{S_{\cdot t}^{(k)},k=1,\cdots,K\right \}$ with the highest importance weights $\left \{w_{t}^{(k)},k=1,\dots,K\right \}$ from the set $\left \{S_{\cdot t}^{(k,l)},w_{t}^{(k,l)},k=1,\dots,K,l=1,\dots,L\right \}$. Normalize the weights of the selected particles. Go back to **Step 2** and repeat until *t*=*n*.
- **Step 4** (Haplotype reconstruction): At *t*=*n*, assemble the entire haplotype sequence by selecting the particle with the largest weight.

#### Complexity analysis of ParticleHap

Since ParticleHap searches for the most likely haplotypes by sequentially extending the partially reconstructed candidate haplotypes one position at a time, it is computationally very efficient and has complexity that scales linearly with the haplotype length, *O*(*n*). On average, the amount of calculations needed for haplotype assembly with ParticleHap is *n* *K* *L*_*a*_ *C*_*a*_, where *C*_*a*_ denotes the average number of bases covering heterozygous sites and *L*_*a*_ denotes the average number of possible extensions of the partially reconstructed haplotype at a heterozygous SNP site. It is worth pointing out that while there are in principle 12 possible SNP pairs of $(s_{l_{1}},s_{l_{2}})$ at one site, the 3^*rd*^ or 4^*th*^ most frequently reported nucleotides are never selected as potential SNPs’ genotypes based on the likelihood calculations. Therefore, ParticleHap can be implemented even more efficiently by retaining the two (or three in the case of ties) most frequently observed nucleotides at each site while the others, which are considered errors, are replaced by −; this step can significantly reduce the amount of likelihood calculations without compromising the accuracy of computing the most likely genotype.

#### Postprocessing

We can further improve the accuracy of assembled haplotypes by pooling the information obtained from multiple runs of ParticleHap whose performance may be affected by the choice of the starting position. In particular, we also run the algorithm in the opposite direction, e.g., perform sequential Monte Carlo reconstruction of the most likely haplotype starting from the site *t*=*n* and terminating the algorithm at the site *t*=1. For the sites where the haplotype pairs reconstructed by the two runs of ParticleHap differ from each other, we compare the likelihoods of the solutions (i.e., we compare the weights in (8)) and choose the one with larger likelihood. In case the sites are consecutive, we compare MEC scores for the two cases and choose the one with smaller MEC.

## Results and discussion

We implemented ParticleHap in C and compared its performance with the publicly available implementations of HapCUT [17] and ReFHap [18]. All three methods are run on a Linux OS desktop with 3.06GHz CPU and 8Gb RAM (Intel Core i7 880 processor). Both real and simulated data are used for the experiments, as described in the remainder of this section.

### genome project data

We first study the performance of ParticleHap on 454 sequencing data of CEU NA12878 genome (1000 Genomes Pilot Project [2]). The short-read data aligned with respect to a reference genome as well as variant and genotype calls for the individual are provided. The data contains a total of 2.58 million reads covering 1.65 million variants on 22 chromosomes. Due to the short lengths of reads and limited insert sizes, the data is split into a number of disconnected blocks. We use ParticleHap to reconstruct each such block.

To evaluate the performance of haplotype assembly, we adopt three measures: the number of phased SNPs (nPhased), the minimum error correction (MEC) score, and running time (Time). In particular, nPhased is the number of SNPs phased by a haplotype assembler; MEC score is the smallest number of entries in the data matrix which need to be changed so that the sequencing information is consistent with an error-free haplotype pair (we report the total MEC score evaluated as the sum of the MEC scores obtained for each haplotype block); and, Time is the runtime of an algorithm in seconds, measured for each algorithm on the same processor.

We choose a different number of particles *K* for each block. Specifically, the longer the block length *n*, the larger the number of particles (12 for *n*≤12, $\frac {n}{2}$ for 12<*n*<100, and 50 for *n*≥100). We assume that the bases in the sequence are equally likely, i.e., the composition probabilities of the 4 nucleotides are equal, 0.25. Note that we assume the error rate *e*=0.01, which is consistent with the typical sequencing accuracy of the 454 platform. Then, the sequencing error probabilities $\phantom {\dot {i}\!}\text {Pr}(X_{\textit {ij}}=x_{\textit {ij}} | S_{\textit {kj}}=s_{l_{k}}) = e/3$ if $\phantom {\dot {i}\!}x_{\textit {ij}}\neq s_{l_{k}}$ and are equal to 1−*e* otherwise.

Table 1 shows that ParticleHap can assemble more haplotypes with higher accuracy and better computational efficiency than HapCUT and ReFHap (the lower the MEC score, the better the performance). Clearly, more SNP sites are phased by ParticleHap than by either HapCUT or ReFHap (please see columns 2, 5 and 8). This can be attributed to the fact that ParticleHap processes reads containing actual base calls (i.e., the reads represented by A, C, G, T and −, rather than the same reads being represented by their binary post-genotype calling counterparts), thus allowing more than two nucleotides at a site, while HapCUT and ReFHap discard some information in the process due to relying on the simplified representation of the reads (via the ternary alphabet with elements 0, 1 and −). It turns out that roughly 1.2−1.5 *%* of the heterozygous positions in the dataset are either tri- or tetra-allelic SNP sites. As can be seen from columns 3, 6 and 9, ParticleHap outperforms HapCUT and ReFHap by consistently achieving lower MEC scores on most of the chromosomes. Note that the MEC scores are calculated only for the haplotype pairs phased by an algorithm; thus, the lower MEC of ReFHap does not necessarily imply that it achieves better performance since it may actually be phasing a smaller number of SNP sites. ParticleHap simultaneously provides longer lengths of phased haplotypes as well as lower MEC scores, demonstrating the high accuracy of the proposed algorithm (note that the total number of reads and allele calls involved in the MEC calculation of ParticleHap is larger than those for HapCUT and ReFHap). This can be partly attributed to the fact that ParticleHap works with genotype likelihoods and allows thorough examination of tri- or tetra-allele SNP sites, which leads to improved genotype accuracy in situations where there are potential errors in “hard” genotype calls used by the competing methods. It is also worth pointing out that ParticleHap is designed to sequentially find the maximum-likelihood solution to the haplotype assembly problem rather than to optimize the MEC criterion while HapCUT uses MEC as its optimization objective (the MEC score is only used to correct potential errors in ParticleHap’s post-processing step); therefore, the superior MEC performance of ParticleHap demonstrates the robustness of the approach. Finally, as reported in columns 4, 7 and 10, ParticleHap assembles haplotypes significantly faster than either HapCUT or ReFHap. In particular, ParticleHap can complete haplotype assembly for each of the 22 chromosomes within 6 seconds, while HapCUT and ReFHap require 59 and 14 seconds for the same task, respectively.

**Table 1:** The performance comparison on a CEU NA12878 data set sequenced using the 454 platform in the 1000 Genomes Project

	ParticleHap			HapCUT			ReFHap		
chr	nPhased	MEC	Time(s)	nPhased	MEC	Time(s)	nPhased	MEC	Time(s)
1	66661	2045	1.07	66616	2293	28.03	66490	2111	5.79
2	78002	2742	1.11	77970	2857	35.71	77853	2698	7.78
3	66217	2111	3.96	66178	2349	29.50	66071	2203	6.20
4	69939	2386	3.94	69901	2591	37.16	69786	2410	9.01
5	63723	1971	4.75	63693	2156	28.06	63605	2044	6.00
6	69750	3312	5.25	69706	3544	58.60	69580	3318	13.39
7	54330	1867	3.31	54302	2059	27.23	54202	1908	7.19
8	56406	1700	3.89	56382	1828	25.87	56281	1690	5.60
9	42244	1335	2.15	42230	1472	20.02	42157	1365	4.52
10	50022	1618	2.73	49998	1814	23.44	49900	1662	5.22
11	46141	1411	2.72	46124	1569	21.66	46051	1467	4.89
12	43333	1467	2.32	43315	1581	20.00	43251	1495	3.92
13	36952	1286	0.68	36937	1398	18.80	36872	1311	7.10
14	30349	887	0.38	30334	982	13.25	30293	916	2.90
15	26626	975	0.88	26614	1055	11.46	26567	968	2.60
16	31675	1156	0.87	31662	1257	14.84	31612	1185	3.98
17	21054	1206	0.59	21048	1223	11.19	21010	1172	5.35
18	28784	851	0.37	28769	936	11.94	28717	855	2.67
19	17018	653	0.25	17006	761	8.35	16961	687	4.25
20	21679	737	0.43	21673	790	9.50	21635	735	2.84
21	14737	485	0.41	14736	525	6.82	14714	500	2.09
22	12929	388	0.28	12925	433	5.38	12891	395	1.74

A comparison of the number of phased SNPs(nPhased), the MEC scores(MEC) and running time(Time) for different haplotype assembly algorithms, ParticleHap, HapCUT and ReFHap, on all of 22 chromosomes

### Simulated data

We further test the performance of our proposed method on the simulated data set. In particular, we examine how the genotype calling errors affect the performance of haplotype assembly. The data are generated using a similar strategy to the one in [15] except that a genotype calling error is included in our simulation data. We generate a pair of phased heterozygous SNP sequences of length *n*, which have genotype calling errors with probability *g*_*e*_. The parameter *g*_*e*_ is judiciously chosen as 0.04 and 0.08 in order to emulate practical scenarios reported in [1] (there, the genotype call accuracy for high call rates was up to 96 % with the use of LD information, from 78 % and 87 % with the use of single sample and multiple individuals, respectively, for the 62 CEU individuals). The true haplotype sequences are generated by emulating genotyping errors; each base in the erroneous heterozygous SNP sequences is flipped to one of the other three nucleotides with equal probability, *g*_*e*_/3. To generate the observation data matrix, instead of sampling (with reads) from true haplotype sequences *c* times as in [15], we sample true haplotype sequences $\frac {c}{2}$ times and sequences containing genotype calling errors $\frac {c}{2}$ times. Each replicate is randomly partitioned into non-overlapping fragments of length between 3 and 7 (the lengths typical of benchmarking data sets in [15]). In order to simulate paired-end (or mate-pair) sequences, we randomly merge some of the generated fragments (fragments whose SNPs are in the first half of haplotype sequence are merged with those whose SNPs in the last half of sequence; as a result, half of the fragments in the dataset are paired-end sequences). Once the fragments are arranged in a SNP matrix, we emulate sequencing errors by randomly flipping a base to one of the other three nucleotides with equal probability. The probability that each base is flipped is 0.03 and 0.01 for *g*_*e*_=0.04 and *g*_*e*_=0.08, respectively, and thus the total error rate for the entries in the SNP matrix is *e*=0.05. To explore the performance of the algorithm over a broad range of experimental parameters, we generate datasets with different SNP lengths (*n*=100, 200 and 300) and vary the coverage rate (*c*=4, 6, 8 and 10) for each genotype calling error rate (*g*_*e*_=0.04 and *g*_*e*_=0.08). For each of the 24 combinations of the parameters, the experiment is repeated 100 times and the results averaged over the 100 instances are reported for each case.

We quantify the ability of an algorithm to reconstruct a haplotype by means of the reconstruction rate [15] defined as

$${}R=\!1-\frac{\min(D(h_{1},\hat{h}_{1})+D(h_{2},\hat{h}_{2}),D(h_{1},\hat{h}_{2})+ D(h_{2},\hat{h}_{1}))}{2l}, $$

where (*h*_1_,*h*_2_) is the pair of true haplotypes, $(\hat {h}_{1},\hat {h}_{2})$ is the pair of reconstructed haplotypes, $D(h_{i},\hat {h}_{j})=\sum \limits _{k=1}^{n} d(h_{i}[k],\hat {h}_{j}[k])$ is the generalized Hamming distance between *h*_*i*_ and $\hat {h}_{j}$, and $d(h_{i}[k],\hat {h}_{j}[k])= 0$ if $h_{i}[k]=\hat {h}_{j}[k]$ and is 1 otherwise. Running time (Time(s)) for each algorithm is evaluated along with the reconstruction rate (ReconRate). In addition, we report the rate at which ParticleHap infers true genotypes for the locations where genotype calling errors are induced, i.e., the improvement rate of genotyping accuracy (labeled as ImpGeAc).

Tables 2 and 3 compare the results of ParticleHap, HapCUT and ReFHap for *g*_*e*_=0.04 and *g*_*e*_=0.08, respectively. As can be seen in those tables, ParticleHap assembles haplotypes with the reconstruction rates of 97.85 % and 95.68 % when the data is affected by the genotype calling error rates of 4 % and 8 %, respectively. This highly accurate performance is achieved in part due to ParticleHap’s ability to improve genotyping accuracy in mis-called (or uncertain) sites by more than 50 % in all the considered scenarios as shown in column 3 in Tables 2 and 3. (i.e, ParticleHap can improve the genotype accuracy of 96 % and 92 % to 98 % and 96 % in Tables 2 and 3, respectively.) It is worth pointing out that, in these simulations, we assumed equal prior probabilities of all genotypes. Imposing more judicious choices of priors may lead to further improvement of genotyping accuracy. On another note, the corresponding reconstruction rates of HapCUT and ReFHap do not exceed 96 *%* and 92 *%*, respectively. Evidently, incorporation of genotyping in the haplotype assembly procedure allows pushing the limits of the achievable accuracy of haplotype assembly. Note that ParticleHap is consistently much faster than HapCUT and ReFHap in all the considered scenarios. As expected, the running time of ParticleHap increases with both the haplotype length and sequencing coverage.

**Table 2:** The performance comparison on a simulated data set for *g* *e*=0.04

	ParticleHap			HapCUT		ReFHap		
n	c	ImpGeAc	ReconRate	Time(s)	ReconRate	Time(s)	ReconRate	Time(s)
100	4	0.6254	0.9785	0.02	0.9598	0.66	0.9497	0.11
	6	0.6252	0.9794	0.02	0.9570	0.84	0.9481	0.25
	8	0.5977	0.9792	0.03	0.9590	1.01	0.9524	0.56
	10	0.5737	0.9780	0.03	0.9582	1.17	0.9517	1.23
200	4	0.5935	0.9779	0.07	0.9594	1.78	0.9499	0.26
	6	0.5977	0.9783	0.08	0.9597	2.26	0.9518	0.88
	8	0.5840	0.9757	0.09	0.9596	2.71	0.9524	2.56
	10	0.5758	0.9777	0.11	0.9593	3.13	0.9528	5.80
300	4	0.6013	0.9715	0.17	0.9591	3.39	0.9493	0.53
	6	0.5848	0.9720	0.20	0.9596	4.34	0.9511	2.12
	8	0.5842	0.9695	0.22	0.9598	5.14	0.9525	6.35
	10	0.5671	0.9703	0.24	0.9599	5.90	0.9537	14.68

A comparison of reconstruction rate(ReconRate) and running time(Time) for different haplotype assembly algorithms, ParticleHap, HapCUT and ReFHap, on the simulated data for *g* *e*=0.04. For ParticleHap, the improvement rates of genotyping accuracy (ImpGeAc) are also reported

**Table 3:** The performance comparison on a simulated data set for *g* *e*=0.08

	ParticleHap			HapCUT		ReFHap		
n	c	ImpGeAc	ReconRate	Time(s)	ReconRate	Time(s)	ReconRate	Time(s)
100	4	0.6211	0.9618	0.02	0.9193	0.66	0.9009	0.11
	6	0.5941	0.9610	0.02	0.9184	0.86	0.8997	0.25
	8	0.5970	0.9585	0.03	0.9184	1.04	0.9017	0.56
	10	0.5845	0.9572	0.03	0.9193	1.19	0.9041	1.26
200	4	0.6262	0.9615	0.08	0.9186	1.80	0.8979	0.27
	6	0.5938	0.9418	0.09	0.9198	2.30	0.9021	0.89
	8	0.6050	0.9389	0.10	0.9193	2.75	0.9019	2.53
	10	0.5997	0.9438	0.12	0.9193	3.16	0.9039	5.84
300	4	0.6245	0.9432	0.18	0.9197	3.43	0.8995	0.53
	6	0.6069	0.9397	0.21	0.9198	4.49	0.9009	2.11
	8	0.6058	0.9315	0.23	0.9186	5.54	0.9009	6.29
	10	0.5792	0.9192	0.27	0.9187	6.37	0.9029	15.00

A comparison of reconstruction rate(ReconRate) and running time(Time) for different haplotype assembly algorithms, ParticleHap, HapCUT and ReFHap, on the simulated data for *g* *e*=0.08. For ParticleHap, the improvement rates of genotyping accuracy (ImpGeAc) are also reported

## Conclusions

In this paper, we presented a novel deterministic sequential Monte Carlo (i.e., particle filtering) algorithm for solving the haplotype assembly problem. ParticleHap sequentially infers the haplotype sequence, one SNP site at a time, by exhaustively searching for the most likely extension of the partially assembled haplotype in each step, examining both the possible genotypes and phase. We tested the performance of ParticleHap on 1000 Genomes Project data, showing that it achieves better minimum error correction scores and phases more heterozygous sites than two of the most accurate existing methods while being significantly more computationally efficient. The results of testing ParticleHap on the simulated dataset also demonstrate that the proposed method can reconstruct haplotypes with higher accuracy and efficiency than those of competing techniques over a wide range of the haplotype assembly problem parameters.

The main goal of ParticleHap is accurate haplotype assembly rather than genotype calling. However, methods that can improve accuracy of genotype calling can be incorporated in the proposed algorithm. For example, the prior information about allele and genotype frequencies or linkage disequilibrium patterns can be incorporated in the proposed algorithm, which may further improve the accuracy of genotype calling and thus of haplotype assembly. In conclusion, the proposed method provides a framework for joint genotyping and haplotyping that leads to accurate haplotype assembly.
